# Solid-State Anaerobic Digestion of Organic Solid Poultry Waste for Biomethane Production

**DOI:** 10.3390/bioengineering12070712

**Published:** 2025-06-29

**Authors:** Faryal Fatima, Raghava R. Kommalapati

**Affiliations:** 1 Center for Energy and Environmental Sustainability, Prairie View A&M University, Prairie View, TX 77446, USA; fatimafaryal.96@gmail.com; 2 Department of Civil and Environmental Engineering, Prairie View A&M University, Prairie View, TX 77446, USA

**Keywords:** solid-state anaerobic digestion, organic solid poultry waste, biodegradability, substrates to inoculum, pH, mesophilic, thermophilic

## Abstract

This study examines biodegradability (BD) and optimum conditions for the solid-state anaerobic digestion (SS-AD) of organic solid poultry waste (organs, intestines, offal, and unprocessed meat) to maximize biomethane production. Three main parameters, substrate-to-inoculum (S/I) ratio, pH, and temperature, were evaluated for the SS-AD of organic solid poultry waste. pH was evaluated at non-adjusted pH, initially adjusted pH, and controlled pH conditions at a constant S/I ratio of 0.5 and temperature of 35 ± 1 °C. The S/I ratios were examined at (0.3, 0.5, 1, and 2) at a controlled pH of ≈7.9 and temperature of 35 ± 1 °C. The temperature was assessed at mesophilic (35 ± 1 °C) and thermophilic (55 ± 1 °C) conditions with a constant S/I ratio of 0.5 and controlled pH of ≈7.9. The results demonstrate that the highest biomethane production and BD were achieved with a controlled pH of ≈7.9 (689 ± 10 mg/L, 97.5 ± 1.4%). The initially adjusted pH (688 ± 14 mg/L, 97.3 ± 1.9%) and an S/I ratio of 0.3 (685 ± 8 mg/L, 96.8 ± 1.2%) had approximately equivalent outcomes. The thermophilic conditions yielded 78% lower biomethane yield than mesophilic conditions. The challenge of lower biomethane yield under thermophilic conditions will be resolved in future studies by determining the rate-limiting step. These observations highlight that SS-AD is a promising technology for biomethane production from solid organic poultry waste.

## 1. Introduction

The consumption of meat has increased with the increase in population. Poultry meat is one of the most consumed meats compared to pork, beef, and others [1]. The Food and Agriculture Organization (FAO) outlook reported that poultry meat is anticipated to represent approximately 41% of all meat protein in 2030 [2]. The shift towards poultry meat consumption in low-income countries is due to the affordable prices, and in developing countries, it is due to its healthy food quality of high protein and lower fat content. It is also reported that from 1990 to 2030, worldwide poultry meat consumption is expected to increase from 152 million tons (MT) [2].

As a result, organic solid poultry waste generated from poultry processing has substantially increased. Organic solid poultry waste is mainly unconsumed and unprocessed meat during slaughterhouse poultry processing. It includes feathers, intestinal residues, blood, bone, feet, offal, and unprocessed meat [3]. They mainly consist of animal protein and fats; thus, they are high in total solids (TS), content of approximately 10–15%. Initially, these wastes were recycled into animal feed through the rendering process or utilized to produce agricultural compost. Currently, the government has imposed various strict regulations to manage this organic waste [3,4].

Other technologies, such as composting, pyrolysis, incineration, and gasification, can manage organic waste from the poultry industry; however, these technologies have high capital and operating costs, energy consumption, emissions, and space requirements, making them invaluable options for waste management [5,6]. Anaerobic digestion (AD) is one of the promising technologies that can manage this waste efficiently by providing material recovery and energy production [7]. In the AD process, the organic waste is broken down in the absence of oxygen by various anaerobic bacteria to produce biogas.

The AD process comprises four main stages: The first stage is hydrolysis, which converts complex organic materials into simpler organic molecules. Acidogenesis is the second stage, involving the breakdown of simple organic and amino acids to volatile fatty acids (VFAs), hydrogen gas (H_2_), and carbon dioxide (CO_2_). Acetogenesis, the third stage, transforms VFA to acetic acid (CH_3_COOH). Methanogenesis, the last stage, uses the by-products of the first two processes to produce biogas, and the remaining residue is called digestate [8].

Biogas and digestate are the two main by-products of the AD process. Biogas consists of 60–70% methane (CH_4_), 30–50% of CO_2_, and traces of other gases, such as hydrogen sulfide (H_2_S) and water vapor (H_2_O) [9]. Biomethane can be used as a renewable fuel for operating vehicles [9], thus replacing fossil fuels, and it can also be converted to electrical and thermal energy. Digestate, which is high in nitrogen content, can be used as a natural fertilizer, replacing synthetic nitrogen fertilizers in the agricultural industry to improve soil fertility [9,10]. The production of biogas as a renewable source through AD is highly favorable for environmental benefits, including greenhouse gas reduction and managing waste of various origins compared to fuels obtained from fossil fuels [11]. Biogas mainly reduces greenhouse gas emissions because of its carbon-neutral property. The CO_2_ emitted during the combustion of biogas is a part of the natural carbon cycle process, hence releasing no CO_2_ into the atmosphere [12].

Biogas production through AD is an environmentally friendly method; however, the performance of AD is strongly dependent on its operating parameters. These parameters primarily include temperatures, pH levels, organic loading rates (OLR), hydraulic retention time, substrate composition, inoculum quality, and the carbon-to-nitrogen (C/N) ratio [13,14]. If these operating conditions are poorly regulated, they can lead to reduced biogas yield, process instability, accumulation of VFA, poor microbial activity, inhibition of the process, and other challenges [15,16]. These limitations become more critical for the AD of organic poultry waste due to its rich protein content, which can lead to high ammonia and VFA concentrations during AD processing. Therefore, proper management of these parameters is essential for maximizing biomethane production for the AD process, specifically for organic poultry waste [17].

There are two main types of AD processes: solid-state AD (SS-AD) and liquid AD (L-AD). In SS-AD, the concentration of TS is higher than 15%, and in L-AD, the TS concentration is lower than 15% [10,18]. SS-AD has various advantages compared to L-AD, such as requiring a smaller reactor capacity, using less heating energy, and requiring no processing energy for stirring [18]. Also, the digestate is easy to handle because of its lower water content. However, the SS-AD process also has some disadvantages, including requiring a high amount of inoculum, the difficulty in operating SS-AD at larger scale, and high capital and operating costs. Ongoing research aims to resolve these challenges.

In this research, the biodegradability (BD) of organic solid poultry waste in SS-AD was evaluated at various substrate-to-inoculum (S/I) ratios, pH levels, and temperature conditions. The main objective of this study was to improve BD and choose the optimum conditions for SS-AD of organic solid poultry waste, which can produce the highest biomethane levels. To our knowledge, past research for organic solid poultry waste has not been conducted in SS-AD. Most of the research has been performed only on L-AD and poultry litter. The novelty of this paper lies in that this study focuses on producing biogas using the SS-AD technology by consuming organic solid poultry waste, other than poultry litter, which has gained limited attention in previous research work. This study opens doors for researchers to conduct further investigations and improve the SS-AD process for organic solid poultry waste.

## 2. Materials and Methods

### 2.1. Substrate and Inoculum

This experiment utilized two kinds of feedstock: organic solid poultry waste and inoculum. The poultry waste consisted of organs, intestines, offal, and unprocessed meat collected from a local poultry processing plant in Bryan, Texas. The pretreatment of poultry waste was carried out by drying it at 105 °C in an oven for five days (Thermo Scientific, Waltham, MA, USA). Then, the size of the poultry waste was reduced to less than 2 mm using a mechanized grinder (LHB dual refiner Model 6SM14A). The inoculum was obtained from the Prairie View A&M University (PVAMU) wastewater treatment plant from an aerobic digester that works at mesophilic temperature. The inoculum was degassed until no gas was produced.

The poultry waste and inoculum were characterized for TS, total volatile solids (TVS), and total fixed solids (TFS) using standard method 2540. The poultry waste was also quantified for carbon (C), hydrogen (H), nitrogen (N), sulfur (S), and oxygen (O) content with a Flash 2000 Organic CHNSO Analyzer (Thermo Fisher Scientific, Waltham, MA, USA).

### 2.2. Biomethane Potential Assays

Biomethane potential assays (BMPs) were conducted in a 250 mL serum bottle (Fisher Scientific, Pittsburgh, PA, USA). For all experiments, the OLR rate was 20% (20 g), and the inoculum quantity was adjusted to obtain the desired S/I ratio. In this study, three parameters were optimized, pH, S/I, and temperature, for the SS-AD of organic solid poultry waste.

For the pH parameter, three different conditions were considered. The first one was a non-adjusted pH sample. The non-adjusted pH sample did not contain any buffer solution, and its natural pH turned out to be in the range of (6.0–6.5). The second condition was initially an adjusted pH sample, for which the pH was adjusted to ≈ 7.9 using 1 M sodium hydroxide (NaOH) solution before starting the BMP. The third condition was a controlled pH sample, for which the pH was maintained at ≈ 7.9 by adjusting it every week using 1 M NaOH. The S/I ratio and temperature were kept constant at 0.5 and 35 ± 1 °C, respectively, for pH optimization.

For the examination of the S/I ratios for the SS-AD of organic solid poultry waste, four different S/I ratios was examined, 0.3, 0.5, 1, and 2, at a controlled pH of ≈7.9 and a constant temperature of 35 ± 1 °C.

For temperature optimization, mesophilic (35 ± 1 °C) and thermophilic (55 ± 1 °C) temperatures were evaluated at a constant S/I ratio of 0.5 and controlled pH at ≈ 7.9.

**Figure 1:**
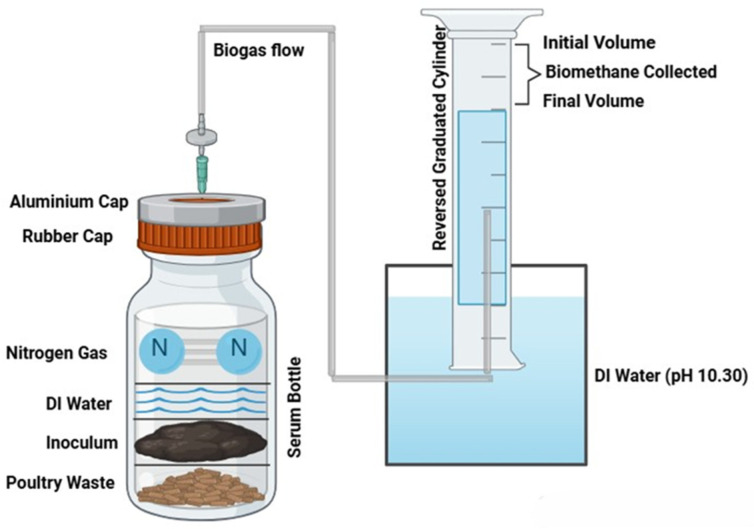
Schematic of liquid displacement method for biomethane measurement.

All experiments were performed in triplicate to validate the results. The working volume of serum bottles was 100 mL. To achieve a total working volume of 100 mL, deionized (DI) water was added to fill the remaining space in the serum bottles. Blank experiments included everything except the substrates. The remaining 150 mL of headspace in the serum bottles was purged with 99% nitrogen gas (N_2_) for 3 min to create an anaerobic environment. The bottles were sealed with rubber plug stoppers and secured with aluminum top crimp seals (Fisher Scientific, Pittsburgh, PA, USA), as shown in Figure 1.

The bottles were shaken manually before and after the daily gas measurement. The data from the experiment were analyzed using Microsoft Excel 2010 (Microsoft, Redmond, WA, USA). Biomethane was measured using the liquid displacement method [19] at ambient conditions. With the liquid displacement method, the pH of the DI water in reversed graduated cylinder and the container holding graduated cylinder was adjusted to 10:30 using 1 M NaOH solution to absorb the CO_2_ and give the estimated biomethane. As stated by Holliger et al. [20], if the liquid displacement method uses an alkaline solution to absorb CO_2_, then direct biomethane values are measured [20]. The biomethane was measured by recording the difference between the initial and final volume, as shown in Figure 1. The final value of the biomethane yield of the sample was calculated by subtracting the biomethane produced from the blank and the sample.

### 2.3. Theoretical Maximum Biomethane Yield

The theoretical maximum biomethane yield (TMY) of poultry waste was determined using Boyle’s Equation below (Equation (1)). Boyle’s Equation is a modification of Buswell Muller’s original equation after including N and S to obtain ammonia (NH_3_) and sulfur dioxide (SO_2_) fractions [21].

(1) $$C_{a}H_{b}O_{c}N_{d}S_{e}+\left(a-\frac{b}{4}-\frac{c}{2}+\frac{3d}{4}+\frac{e}{2}\right)H_{2}O\to \left(\frac{a}{2}+\frac{b}{8}-\frac{c}{4}-\frac{3d}{8}-\frac{e}{4}\right){CH}_{4}+\left(\frac{a}{2}-\frac{b}{4}+\frac{c}{4}+\frac{3d}{8}+\frac{e}{4}\right){CO}_{2}+{dNH}_{3}+{eH}_{2}S$$

(2) $$TMY\;(\frac{mL}{gVS})=\frac{22.4\times 1000\times [\left(\frac{a}{2}\right)+\left(\frac{b}{8}\right)-\left(\frac{c}{4}\right)-\left(\frac{3d}{8}\right)-\frac{e}{4}]}{12a+b+16c+14d+32e}$$

where *a*, *b*, *c*, *d*, and *e* represent the elemental composition coefficients of *C*, *H*, *O*, *N*, and *S*, respectively, in the empirical formula.

### 2.4. Biodegradability

A substrate’s BD indicates the fraction of its volatile solids (VSs) converted into biomethane throughout the AD process. The BD was calculated by dividing the experimental methane yield (EMY) by the TMY and multiplied by 100, as shown below in Equation (3) [21].

(3) $$BD\left(\%\right)=\frac{EMY}{TMY}\times 100$$

## 3. Results

### 3.1. Substrate Characterization

The results for substrate characterization show that organic solid poultry waste consisted of C, H, N, S, and O contents of 60.3%, 9.5%, 6.1%, 0.5%, and 23.6%, respectively, with TSs of 99.0% and TVSs of 98.0%, as shown in Table 1. Thus, this indicates that most of the solids of poultry waste are organic matter. The TSs and TVSs of inoculum were measured as 3.0% of TSs and 1.0% of TVSs. The C, H, N, S, and O contents of inoculum were not measured, as they were in a sludge form that was incompatible with the CHNSO analyzer. The TMY of organic solid poultry waste was calculated as 707 mL/gVS.

From the elemental composition, the C/N ratio for organic solid poultry waste was calculated, resulting in 9.9. In general, for methane production, a substrate with a higher C/N ratio provides more C. On the other hand, a lower N level hinders microbial activity, as bacteria need ample N to sustain their growth [14]. However, researchers have reported various ranges of optimal C/N ratios for biomethane production. A study conducted by Ceron et al. [22] on the effect of the C/N ratio on biogas production from wastewater reported an optimum C/N ratio of 8.2. Moreover, research led by Siddiqui et al. [23] on the co-digestion of industrial food waste and sewage sludge observed that the highest biomethane production was obtained at a 15.0 C/N ratio. Additionally, the study conducted by Wang et al. [24] on the influence of temperature and C/N ratio on the anaerobic co-digestion of dairy manure, chicken manure, and rice straw concluded that by increasing C/N ratios, the reduction in ammonia inhabitation and increase in methane production were achieved with C/N ratios of 25.0 and 30.0 at 35 °C and 55 °C.

To summarize, a suitable range of C/N ratios for biomethane production through an AD process have been reported between 8.0 and 30.0 [22,23,24], and the C/N ratio of 9.9 for poultry organic solid waste falls within that range.

### 3.2. Effect of pH on Daily and Cumulative Biomethane Production

**Figure 2:**
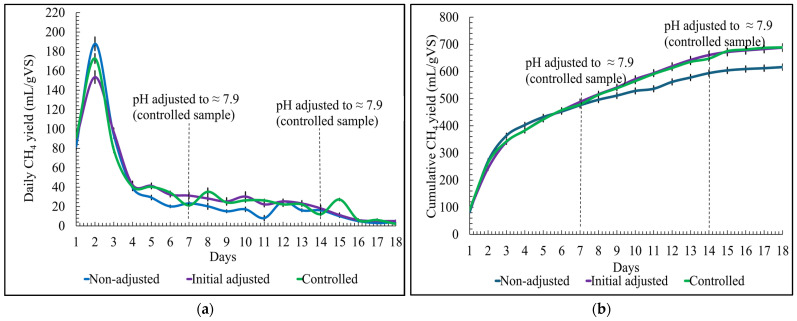
pH optimization for biomethane yield: (**a**) daily; (**b**) cumulative.

The results for daily biomethane production reveal that on day one, the biomethane production started for all the pH conditions, including the non-adjusted pH sample, initially adjusted pH sample, and controlled pH sample, as shown in Figure 2a. The highest peak for biomethane yield for all pH conditions was achieved on the second day. For the non-adjusted pH sample, the biomethane yield on the second day was approximately 188 ± 7 mL/gVS. For the initially adjusted pH sample and controlled pH sample, a peak at 154 ± 7 mL/gVS and 172 ± 5 mL/gVS, respectively, was achieved.

It was observed that biomethane production gradually decreased after the second day. When the pH was adjusted back to 7.9 for the controlled sample on days seven and fourteen, the biomethane production slightly increased on the following day. After the fifteenth day, the total biomethane production was less than 1% of what was previously accumulated for three consecutive days; therefore, the experiment ended by day eighteen.

The hydraulic retention time for the SS-AD of poultry organic solid waste came out to be around eighteen days. The hydraulic retention time results were compared with the research conducted by Salminen et al. [25] on the effect of hydraulic retention time and loading rate on the semi-continuous AD of poultry waste; they observed that as the OLR was increased, the retention time decreased. The shortest retention time of 13–25 days was observed for the semi-continuous AD of poultry waste. In SS-AD, the OLR is higher compared to that under L-AD conditions; therefore, a shorter retention time was observed compared to that in the study conducted by Yoon et al. [3].

The cumulative biomethane yield for the non-adjusted pH sample was the lowest at approximately 616 ± 11 mL/gVS, while the initially adjusted pH sample was the second highest at 688 ± 14 mL/gVS, and the controlled pH sample was the highest at 689 ± 10 mL/gVS, as shown in Figure 2b. The cumulative biomethane yield of the initially adjusted pH sample and controlled is almost equal. This could be because when the sample for controlled pH was checked before setting it back to a pH of 7.9, the pH did not drop significantly; the pH was at around 7.5. Thus, this shows that the pH at 7.5 is also favorable for the SS-AD of organic solid poultry waste. However, the pH under the neutral condition (in the range of 6.0–6.5) was not favorable for the SS-AD of organic solid poultry waste. The results achieved in this study confirm the findings outlined by Ajayi et al. [14] in their examination of parameters influencing SS-AD; they concluded that the optimum pH condition to produce biomethane via SS-AD requires maintaining pH in the range of 7.5–8.3.

### 3.3. Effect of S/I Ratios on Daily and Cumulative Biomethane Production

**Figure 3:**
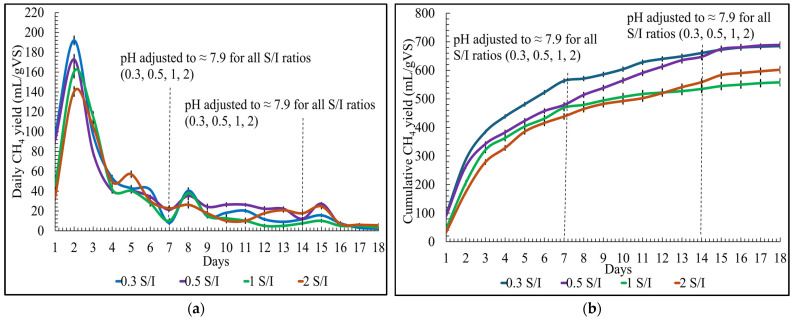
S/I ratio optimization for biomethane yield: (**a**) daily; (**b**) cumulative.

For the S/I ratios, the daily biomethane yield on day one was approximately 96 ± 2 mL/gVS, 91 ± 4 mL/gVS, 45 ± 4 mL/gVS, and 33 ± 3 mL/gVS for 0.3, 0.5, 1, and 2, respectively. Similarly to the pH conditions, the highest biomethane yield was produced on the second day for all S/I ratios, as shown in Figure 3a. It was observed that as time passed, the biomethane production patterns were similar for all S/I ratios. Adjusting the pH weekly slightly improved the daily biomethane yield on the following day. The hydraulic retention time for the S/I ratios was also eighteen days.

The cumulative biomethane yield for S/I ratios showed that the biomethane yield was the lowest 558 ± 13 mL/gVS for the S/I ratio of 1 and the highest 689 ± 10 mL/gVS for the S/I ratio of 0.5, as shown in Figure 3b. Also, the cumulative biomethane yield for 0.3 S/I ratio was close to a ratio of 0.5 S/I, approximately 685 ± 8 mL/gVS. This could be due to the fact that following a particular substrate concentration, microbial activity is not significantly improved. Also, the 4 mL difference could be ignored due to human error in measurement; in reality, the values would be very close.

The cumulative biomethane yield for the S/I ratio of 2 was around 602 ± 12 mL/gVs, which was higher than the S/I ratio of 1, which means at the S/I of 2, the microbial activity was better than the S/I of 1. The S/I ratio below 1 is highly favorable for the SS-AD of organic solid poultry waste. The results achieved in this study correspond to the results obtained by Jansson et al. [26] on the AD of food waste and paper waste. In their study, an S/I of 0.5 achieved the highest biomethane production for both wastes, and increasing S/I from 0.5 to 1 was not favorable for methane production. Also, the higher ratio led to the accumulation of VFA, causing process instability.

In another study conducted by Renggaman et al. [27] on the effect of S/I ratios and inoculum type on the BMP of solid agro-industrial waste, they evaluated the S/I ratios of 0.25, 0.5, 1, and 2, and their results showed that optimum S/I ratios for the methane potential of the agro-industrial waste were 0.5 and 0.25, respectively. A higher S/I ratio delayed methane production, indicating process inhibition.

### 3.4. Effect of Temperature on Daily and Cumulative Biomethane Production

For the SS-AD of organic solid poultry waste, two different temperature conditions were tested, mesophilic (35 ± 1 °C) and thermophilic (55 ± 1 °C), at a constant S/I ratio of 0.5 and controlled pH at 7.9. The daily biomethane yield results show that as the temperature increased, the daily biomethane yield decreased. Daily biomethane yield was lowest for thermophilic conditions each day; it started with a daily peak of 23 ± 3 mL/gVS on day one. However, for mesophilic conditions, the daily biomethane yield started at a peak of 91 ± 4 mL/gVS.

**Figure 4:**
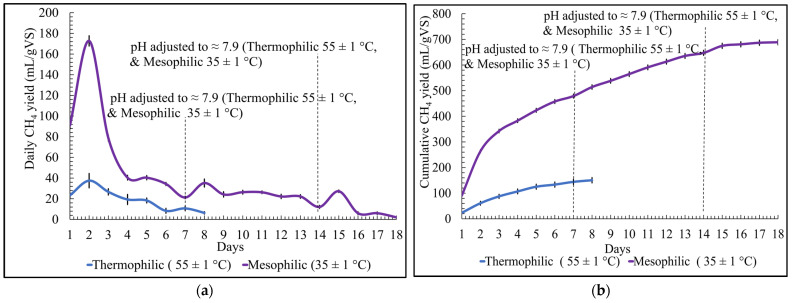
Temperature optimization for biomethane yield: (**a**) daily; (**b**) cumulative.

On the second day, at the mesophilic temperature, the biomethane yield was approximately 4.5 times higher than the thermophilic temperature, which was 38 ± 7 mL/gVS, as shown in Figure 4a. The hydraulic retention time for thermophilic conditions was 10 days shorter than for mesophilic conditions. The cumulative biomethane yield for thermophilic conditions was 150 ± 10 mL/gVS, which was 78% lower than the cumulative biomethane yield under mesophilic conditions, which was 689 ± 10 mL/gVS, as shown in Figure 4b. One of the reasons that the thermophilic temperature produced less biomethane than at the mesophilic temperature is that at the same total ammonia levels, thermophilic conditions face much higher free ammonia stress [28]. Furthermore, organic poultry waste, being high in N content releases free ammonia during digestion [17], may lead to the inhibition and inefficiency of the process at thermophilic temperatures compared to mesophilic temperatures.

The results achieved in this study correspond to the results achieved by Bi et al. [29] on the evaluation of OLR on AD of the poultry manure under mesophilic and thermophilic temperatures. They observed that less biomethane was produced under thermophilic conditions compared to at the mesophilic temperature. The reason stated by Bi et al. [29] in their study was that under thermophilic conditions, the higher loading rate led to free ammonia concentration, which resulted in a high concentration of VFA, thus negatively impacting the AD process. Moreover, the research conducted by Bi et al. [30] on comparing the mesophilic and thermophilic AD of food waste concluded that the thermophilic condition produced a 5% higher biomethane yield than at the mesophilic temperature. The results achieved by Bi et al. [30] disagree with the results obtained in this study because the thermophilic temperature produces a 78% lower biomethane yield compared to the mesophilic temperature for the SS-AD of organic solid poultry waste. It is highly recommended that detailed research be conducted on evaluating mesophilic and thermophilic conditions for the SS-AD of organic solid poultry waste to fully understand the underlying reason for the significant difference in biomethane yield.

### 3.5. Biodegradability

**Figure 5:**
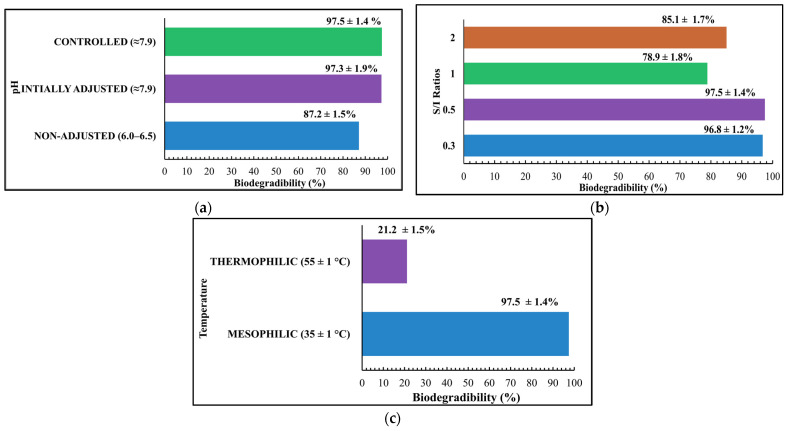
Biodegradability of organic solid poultry waste: (**a**) pH; (**b**) S/I ratio; (**c**) temperature.

For the pH conditions, the highest BD of organic solid poultry waste was achieved for the controlled pH (7.9) sample of 97.5 ± 1.4%. The BD of the initially adjusted pH (7.9) sample was close to the controlled pH sample of 97.3 ± 1.9%. The lowest BD of 87.2 ± 1.5% was obtained for the non-adjusted pH sample, as shown in Figure 5a. The AD process is highly sensitive to pH parameters; therefore, maintaining the appropriate pH condition is very important for the AD to produce a higher biomethane yield and to protect it from digestive failure [18]. The BD of the non-adjusted pH sample was the lowest. This can be explained by the fact that as in the non-pH adjusted sample, the natural pH range was between 6.0 and 6.5, and in the AD process, the acetogenic bacteria and methanogenic archaea thrive at the pH range of 6.6–7.7 and 7.5–8.5, respectively [8]. Since the pH of the non-adjusted sample is lower than this range, VFAs were accumulated and they inhabited these last two stages, which resulted in lower methane yield and, thus, lower BD.

For the S/I ratios, the highest BD was achieved for the 0.5 S/I ratio of 97.5 ± 1.4%, the BD for 0.3 S/I ratio was very close to the 0.5 S/I ratio around 96.8 ± 1.2%, the second lowest BD of 85.1 ± 1.7% was achieved for the S/I ratio of 2, and the lowest BD of 78.9 ± 1.8% was achieved for the S/I ratio of 1, as shown in Figure 5b. This can be explained by Li et al.’s [31] study on the relationship between the effects of the S/I ratio with microbial communities during the AD of food waste. The results showed that high S/I ratios (4:1, 3:1, 2:1, and 1:1) were affected by irreversible acidification. However, the low S/I ratios (1:2, 1:3, and 1:4) favored methanogenesis activity. The structure analysis of the microbial community in the AD process revealed that as the S/I decreased, the abundance and diversity of bacteria and archaea, specifically synergistetes and Bacteroidetes, increased, which helped in producing higher biomethane yield [31].

The same underlying reason justifies the results obtained in this study in that higher BD was achieved for the lower S/I (0.3, 0.5) ratios; at low S/I ratios, the abundance of the microorganisms increased, hence sustaining methane production. Moreover, at high S/I ratios (1, 2), the accumulation of VFA in the SS-AD process occurred, leading to the inefficiency and failure of the SS-AD process.

For temperature conditions, the BD achieved at thermophilic temperature was 21.2 ± 1.5%, and 97.5 ± 1.4% for the mesophilic temperature, as shown in Figure 5c. The lower BD for the thermophilic temperature condition can be justified by the research conducted by Kim et al. [32], which investigated the effects of mesophilic and thermophilic temperature on the performance and microbial activity in the AD of food waste. The study highlights that a higher biomethane yield was achieved under mesophilic conditions compared to thermophilic temperature because a substantial accumulation of propionate and acetate acid was found in the thermophilic temperature, indicating that the syntrophic acetogenic bacteria (SAB) were not efficiently working in the acetogenesis step. However, low levels of propionate and acetate acids were observed for the mesophilic condition, indicating the stability of acetogenesis and methanogenesis and consequently improving the overall biomethane yield and, hence, BD [32].

In this study, a similar explanation can be applied that under thermophilic conditions, VFA accumulated during the acetogenesis step because the acetogenic bacteria were not effectively converting VFA into intermediate products (CH_3_COOH, H_2_, and CO_2_), which eventually converts to methane in the methanogenesis stage. Consequently, a lower BD yield was achieved under thermophilic conditions.

## 4. Conclusions

It is concluded that pH, S/I ratio, and temperature can significantly influence biomethane yield for the SS-AD of organic poultry waste. The experimental findings show that the optimum condition to achieve the highest BD for the SS-AD of organic poultry waste was within a controlled pH of 7.9 at an S/I of 0.5 under mesophilic conditions. It was observed that after reaching a certain substrate concentration and pH levels, the biomethane yield does not improve considerably. Specifically, the BD achieved at an S/I of 0.3 and the initially adjusted pH sample was very close to that under the optimum conditions. Additionally, the thermophilic conditions were found to be unfavorable for maximizing the biomethane yield. Overall, the findings reveal that SS-AD is a potential method for producing biomethane for organic poultry waste. It is highly recommended to researchers that future studies be performed on various parameters, such as OLR, hydraulic retention time, co-digestion, and others that can influence and improve biomethane production through the SS-AD of organic solid poultry waste. In parallel, our future studies will focus on optimizing temperature ranges and C/N ratios for the SS-AD of organic poultry waste. In addition, comprehensive studies will be conducted on microbial activity, ammonia concentration, VFA levels, and digestate quality to further improve the SS-AD process for organic poultry waste. Further work would be needed to improve the biomethane yield for the SS-AD of poultry waste under thermophilic conditions. To achieve this, rate-limiting step at thermophilic temperatures needs to be determined, followed by applying necessary strategies such as co-digestion, pretreatment, cultivation of microbial community, or any applicable techniques.

## Figures and Tables

**Table 1:** Characterization of organic solid poultry waste and inoculum.

Parameter	Poultry Waste	Inoculum
Moisture content (%)	1.0 ± 1.0	96.0 ± 0.2
TS (%)	99.0 ± 1.3	3.0 ± 0.4
VS (%)	98.0 ± 0.5	1.0 ± 0.1
C (%)	60.3 ± 0.4	-
H (%)	9.5 ± 0.5	-
N (%)	6.1 ± 0.2	-
S (%)	0.5 ± 0.7	-
O (%)	23.6 ± 0.3	-
C/N	9.9	-

## Data Availability

Data are contained within the article.

## Abbreviations

AD	Anaerobic digestion
BD	Biodegradability
BMP	Biomethane potential assay
C	Carbon
CH_4_	Methane
CH_3_COOH	Acetic acid
C/N	Carbon-to-nitrogen
CO_2_	Carbon dioxide
DI	Deionized
FAO	Food and Agriculture Organization
H	Hydrogen
H_2_	Hydrogen gas
H_2_O	Water vapor
H_2_S	Hydrogen sulfide
L-AD	Liquid anaerobic digestion
MT	Million tons
N	Nitrogen
N_2_	Nitrogen gas
NaOH	Sodium hydroxide
NH_3_	Ammonia
O	Oxygen
OLR	Organic loading rates
S	Sulfur
SAB	Syntrophic acetogenic bacteria
SS-AD	Solid-state anaerobic digestion
S/I	Substrate-to-inoculum
SO_2_	Sulfur dioxide
TFS	Total fixed solids
TMY	Theoretical maximum biomethane yield
TVS	Total volatile solid
TS	Total solid
VFA	Volatile fatty acid
VS	Volatile solid
